# Overcoming the Limitations of *Operando* Microscopy and Spectroscopy for Revealing Degradation Mechanisms of Electrocatalysts in Liquid Media: Correlative Approaches

**DOI:** 10.1021/acs.nanolett.6c02434

**Published:** 2026-07-29

**Authors:** Andreas Hutzler, Siowwoon Ng, Johannes Will

**Affiliations:** † Helmholtz Institute Erlangen-Nürnberg for Renewable Energy (IET-2), 28334 Forschungszentrum Jülich GmbH, Cauerstraße 1, 91058 Erlangen, Germany; ‡ Department of Chemistry and Pharmacy, Materials Chemistry Section, 9171 Friedrich-Alexander-Universität Erlangen-Nürnberg (FAU), Egerlandstraße 3, 91058 Erlangen, Germany; § Institute of Micro- and Nanostructure Research (IMN) and Center for Nanoanalysis and Electron Microscopy (CENEM), 9171 Friedrich-Alexander-Universität Erlangen-Nürnberg (FAU), Cauerstraße 3, 91058 Erlangen, Germany

**Keywords:** electrochemistry, liquid-phase electron microscopy, scanning electrochemical probe microscopy, X-ray microscopy, scattering, spectroscopy

## Abstract

*Operando* microscopy methods applied in electrocatalysis come with critical limitations such as statistical relevance, interfering probe–sample interactions, and mass-transport-related phenomena on the one hand. On the other hand, those methods provide unique insights into dynamic processes that nanomaterials undergo during operation, insights that are inaccessible otherwise. Overcoming these challenges is, therefore, essential to disentangle, for instance, degradation mechanisms of electrocatalysts, knowledge that is urgently needed to drive forward our energy transition in a reasonable manner. This Mini-Review names the details of these challenges, provides recently published solutions, and depicts how *operando* microscopies can be embedded into correlative workflows to unfold their full potential. This way, insights gained from investigating model systems employing electrochemical liquid-phase electron microscopy, *operando* X-ray methods, and scanning electrochemical probe microscopies have the potential to lead to major breakthroughs in the development of next-generation energy materials.

## Graphical abstract

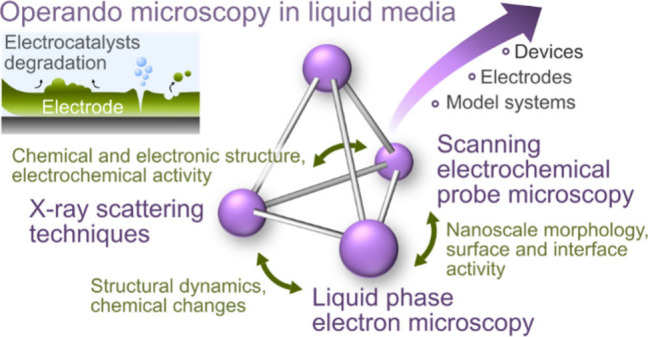

## Introduction

Catalyzed chemical reactions involve highly complex, multiscale mechanisms comprising atomistic processes at interfaces interrelated with macroscopic mass-transport phenomena. This scale-bridging nature of catalysis is one of the main reasons for our limited understanding of underlying processes.^1^ On top, contradicting the initially assumed picture, catalyst surfaces are no static entities undergoing highly volatile surface reconstruction at the atomic scale, which has been proven via *operando* photoemission electron microscopy by Ertl^2^ in 1991. Since then, *operando* gas-phase electron microscopy imaging has been established as a powerful tool for studying heterogeneous catalysis down to the atomic scale.^3^ *Operando* microscopy approaches are, therefore, proven suitable for revealing nanoscale dynamics of catalytic reactions, rendering them invaluable tools in the arsenal of characterization techniques employed nowadays to move beyond the iterative and trial-and-error-based catalyst development. Nevertheless, as inherent for microscopy-based approaches, *operando* microscopy comes along with statistical issues related to sample size. As microscopy in general is not good in statistics, embedding into correlative multiscale and multimodal characterization workflows is the key. Doing so promises to unambiguously link the nanostructure of electrocatalysts and changes they undergo with device-scale characteristics. Such information is urgently needed to accelerate the development of efficient catalysts, required for our fast energy transition toward renewable resources.^1^

While heterogeneous catalytic processes in the gas phase can be sufficiently well analyzed employing such correlative workflows, most electrocatalytic reactions involve liquid phases. This may sound like a marginal difference, because a liquid phase in a crude approximation is just a denser molecular ensemble compared to a gas phase. Nonetheless, this comes along with some critical limitations of *operando* microscopy approaches for studying electrocatalytic processes, which are

i) The probability of probe–sample interactions massively increases, causing the observation method itself to get involved in observable processes (e.g., via radiolysis as the most infamous example).
ii) Such interactions not only interfere with processes to be observed but also limit the resolution capability of *operando* microscopy.
iii) Due to small sample geometries required for many setups, mass-transport-related phenomena may be more pronounced.
iv) The applicability of downstream chemical product or dissolution analysis methods such as mass spectrometry is limited.

This Mini-Review, therefore, gives an overview of recent developments and advancements in *operando* microscopy modalities. Their application in electrocatalysis reveals degradation mechanisms across scales from atomistic scales toward the ensemble level. This knowledge is urgently needed to support the acceleration of catalyst development for driving the energy transition forward. Herein, we specifically present liquid-phase electron microscopy (LP-EM), *operando* X-ray approaches, and scanning electrochemical probe microscopy (SEPM) methods as suitable approaches. Alongside recapitulating recent advancements, we show how these methods push the boundaries forward rendering those finally suitable for quantitative analyses, enhancing the significance of correlative materials research.

## Liquid-Phase Electron Microscopy

Liquid-phase electron microscopy was first conceptualized during the early years of transmission electron microscopy in 1944 by Abrams and McBain.^4^ Its technical implementation, however, took almost 60 years until Williamson *et al.* were finally able to employ semiconductor technology for fabricating liquid cells, small containers encapsulating liquid specimens compatible with the high vacuum of transmission electron microscopes.^5^ Although seminal publications about growth and degradation of nanomaterials have given unprecedented insights into atomistic processes, it became clear very quickly that the electron beam heavily influences these observations. In 2014, Schneider *et al.* transferred knowledge from nuclear research to LP-EM, introducing a physicochemical simulation method to the community that is suitable for estimating the changes in solution chemistry of water upon inelastic scattering of high-energy electrons.^6^ This process denoted as radiolysis has already been compared by Marie Skłodowska Curie to an electrolysis without electrodes and describes the decomposition of a liquid upon energy transfer from ionizing radiation of any kind.^7^ Meanwhile, TEM liquid holder systems are nowadays commercialized offering capabilities beyond encapsulating static liquids only. For instance, *operando* heating or biasing capabilities have been successfully integrated into silicon-chip based liquid cells utilizing miniaturized coplanar electrodes. Moreover, engineering of mass transport in liquid cells such as chips providing high flow rates,^8^ an unidirectional liquid flow across the viewing window,^9^ or monolithically integrated liquid cells with almost no window bulging^10^ enabled fundamental studies on beam-sample interactions and more deliberate experimental design.

Even though these seminal methodological advancements enhancing our knowledge about the nanoworld tremendously, the general opinion about LP-EM as a useful tool remained very limited, unanimously reflected in reviews in the field naming three main issues to be resolved:

i) to deal with radiolysis reliably,
ii) to enhance the resolution capability in LP-EM, and
iii) to overcome the very limited access to chemical information.^11–13^

In fact, today these above-mentioned issues have actually been largely resolved, and new avenues to apply LP-EM in electrocatalysis for generating knowledge have been opened by a huge collective effort of the community. Although previously doubted and often overlooked, quantitative agreements between computational radiolysis modeling and experimental observation have been presented in recent years.^14–17^ In addition, strategies for mitigating beam-sample interactions such as low-dose imaging or sparse scanning have been proposed.^18^ Moreover, modeling input parameters such as the electron inelastic mean free path^10^ or generation values (G-values) of primary radiolysis products^19^ were measured successfully via electron holography or high-resolution electron energy loss spectroscopy (EELS) or are subject to current studies for LP-EM conditions. These methods do not only serve to disentangle and explain processes observed but also have some predictive character allowing for deliberately tuning the solution chemistry to a certain extent.^15^ For example, quantitative agreement between simulated concentrations of observable species (e.g., growth rates of nanostructures) allows for disentangling their reaction pathways.^16^ Nevertheless, it must be accepted that beam-sample interactions can never be fully excluded. Keeping this in mind, parameters such as the reactivity of an aqueous solution by means of its redox potential determined by the concentration of oxidative (e.g., hydroxyl radicals) and reductive species (such as solvated electrons and hydrogen radicals) can be adjusted as well as its acidity (i.e., concentrations of protons and hydroxide ions).^20,21^ This can be achieved by tailoring beam parameters (e.g., probe current, STEM dwell time, beam diameter, etc.) as well as mass transport, e.g., via a liquid flow through the holder system.^15^ In detail, controlling the electron dose rate enables tuning the amount of primary products, whereas tailoring mass transport can be employed to control removal of stable (e.g., molecular H_2_, O_2_, or H_2_O_2_) species.^8,15,17^ Multiphysics modeling together with fundamental parameters recently determined even allows for predicting the spatial distribution of chemical species within a liquid cell reactor,^22^ as well as beam-induced heating,^23,24^ or charging.^25,26^ Indeed, gaining control over the solution chemistry in LP-EM opens new directions for research in electrocatalysis such as accelerated degradation analysis where the radiolysis bug can be used as useful feature instead.^27^ For instance, in PEM water electrolysis, hydroxyl radicals cause membrane decomposition, whereas hydrogen crossover from the cathode is known to enhance iridium dissolution at the anode side.^28^ Both species could be deliberately generated in LP-EM and their concentrations tuned to simulate such degradation for studying underlying microscopic processes. Hereby, molecular hydrogen generation is pronounced at low flow velocities, larger STEM dwell times, and at higher magnification in a spur overlap condition,^17^ whereas hydroxyl radicals could be produced at high dose rates and by adding N_2_O for converting solvated electrons into OH.

In *operando* LP-EM employing electrochemical stimuli via coplanar electrodes integrated into liquid cell chips, mass-transport-related phenomena play an additional role alongside radiolysis. For instance, concentration gradients as well as solution exchange are known to severely influence reference potentials that are of utmost importance for quantitative analyses. In addition, the electrode geometries and materials used for creating reference electrodes are crucial. Therefore, much effort has been put into the development of dedicated electrochemical chips for LP-EM by means of geometry optimization, enhanced mass transport, and more reliable reference electrodes, including internal and external RE setups.^29,30^

Utilizing these recent advancements in LP-EM together with more established state-of-the-art methods such as scanning-flow cells coupled on-line to inductively coupled plasma mass spectrometry (SFC-ICP-MS), identical location electron microscopy, or *operando* X-ray analyses in correlative workflows helps determine inherent uncertainties coming along with LP-EM alone. This enables gaining unprecedented insights into electrochemical processes at the nanoscale.^31,32^ Although LP-EM analyses are limited to model systems, such new insights can help decipher degradation mechanisms in electrocatalysis at a more fundamental level. Such mechanisms are so far inaccessible with any other method, rendering LP-EM as an invaluable extension of our characterization toolbox.

Correlative workflows employing LP-EM could potentially help reveal the underlying processes of degradation phenomena occurring in energy conversion systems such as proton exchange membrane water electrolyzers.^28^ Recent studies have already shown the capabilities and power of LP-EM analyses in revealing degradation or iridium-based electrocatalysts^33,34^ and can now be extended to *operando* LP-EM employing external electrochemical stimuli. Proving correlations between (electro)­chemical signals obtained from LP-EM with those of conventional setups up to the device level can help ensure a statistical relevance of nanoscale observations. This allows for establishing the missing link between atomistic processes and ensemble characteristics in liquid environments.

## Operando X-ray Techniques

Unlike LP-EM, X-ray-based techniques—and in particular high-energy X-ray scattering—are widely established as powerful tools for mitigating key challenges in *operando* characterization.^35,36^ These include bridging the materials gap from well-defined model systems to complex, application-relevant systems, overcoming the pressure gap, accessing time scales ranging from milliseconds to days, and linking structural information from the atomic to the mesoscale.^35,37^ However, one challenging aspect is the presence of beam-induced effects during *operando* studies. Seminal work by Henderson^38^ has shown that for imaging the useful signal per incident photon is orders of magnitude lower than for electrons, due to the reduced ratio of elastic to inelastic scattering events. At the same time, electron beams are well-known to induce substantial radiolysis.

Analogous to electrons, photons interact with matter via both elastic (signal generating) and inelastic processes. Inelastic scattering produces photoelectrons, which can initiate cascades of secondary electrons extending up to tens of micrometers beyond the primary beam path.^35,39^ These processes can generate reactive species that promote radiolysis reactions and locally alter the chemical environment,^14^ for example, by changing the acidity.^20^ Notably, photoelectrons generated within the electrode can reach the electrolyte,^17^ potentially leading to an accumulation of radiolysis products at the electrode–electrolyte interface.^40^ To mitigate such effects, several experimental strategies have been developed, including sample translation, beam spreading, attenuation, and systematic dose variation combined with comparisons between exposed and unexposed regions.^35,36^

Beyond mitigation strategies, quantitative modeling and mechanistic understanding of beam-induced effects are as critical for *operando* X-ray studies as they are in LP-EM. A key parameter enabling such analysis is the dose rate, ψ, which provides a quantitative measure of energy deposition and can be expressed as

$$\psi =\frac{\mu_{en}}{\rho }\Phi E$$

where μ_en_/ρ is the mass-energy absorption coefficient, Φ the photon flux, and *E* the photon energy.

In the hard X-ray regime (10–100 keV), μ_en_/ρ is primarily governed by the photoelectric effect and incoherent (Compton) scattering, accounting for the fraction of photon energy transferred to secondary charged particles as well as subsequent radiative losses. Table 1 summarizes representative dose rates under typical synchrotron conditions (e.g., maximum flux at P08 DESY in focusing mode) for water and iridium, serving as model systems for the electrolyte and electrode, respectively. The data highlight two general trends: (i) a decrease in the dose rate with increasing photon energy, largely due to the reduced photoelectric cross-section, and (ii) significantly stronger energy deposition in high-Z electrode materials compared to the aqueous environment.

**Table 1:** Typical Dose Rates Calculated for Water and Iridium at a Synchrotron Beamline Operated with 10 and 100 keV, Respectively^a^

	Water		Iridium	
*E* [keV]	10	100	10	100
Φ [photons/s/μm^2^]	10^8^			
μ_en_/ρ [cm^2^/g]	4.94	0.025	1.04 × 10^2^	2.1
ψ [Gy/s]	7.9 × 10^4^	4.0 × 10^3^	1.7 × 10^6^	3.4 × 10^5^
ψ [meV/particle/s]	14.8	0.75	3400	680
σ_coh_ [cm^2^/g]	0.23	0.005	4.67	0.19
σ_incoh_ [cm^2^/g]	0.16	0.16	4.70	0.10
σ_ph_ [cm^2^/g]	4.94	0.003	104	4.56

a Data for the mass energy-absorption coefficients as well as the coherent, incoherent and photoelectric absorption cross-section taken from Hubbell and Seltzer.^41^.

Two additional aspects are critical when interpreting these trends. First, the reduction in the dose rate at higher photon energies is accompanied by a decrease in the coherent scattering cross section, which directly impacts the obtainable signal and must be considered when optimizing the photon energy for a given system. Second, increasing the photon energy leads to higher-energy secondary electrons and, consequently, an expanded interaction volume. This results in a spatial redistribution of deposited energy: while the local dose density within the beam footprint decreases, the affected volume extends further into the surrounding material. Together, these effects underscore the importance of carefully balancing photon energy, dose rate, and signal requirements in *operando* X-ray experiments.

A key advantage of high-energy X-rays lies in their large penetration depth, enabling signal collection from substantially larger sample volumes compared with electrons. Nevertheless, careful optimization of irradiation parameters—including photon energy, dose, and dose rate—is essential to balance signal quality against beam-induced artifacts.^35^ In this context, low-background instrumentation with high dynamic range, combined with rigorous data treatment strategies such as denoising^42^ and efficient use of detector counts, becomes critical for reliable *operando* measurements. High-energy X-rays further enable access to extended regions of reciprocal space due to their short wavelength (i.e., large wavenumber). Consequently, the Ewald sphere radius increases, allowing multiple reciprocal lattice points to be sampled simultaneously within a single measurement. Although the Ewald sphere remains comparatively small relative to that in electron diffraction, this limitation is largely compensated for by the wide angular range over which scattered photons can be collected. In contrast to electron diffraction, where the accessible scattering angles are often restricted by instrument geometry, X-ray scattering experiments can capture a broad range of momentum transfers. As a result, both techniques can probe comparable regions of reciprocal space, albeit through fundamentally different scattering geometries and constraints.^43^

Dedicated transmission *operando* cells^36^ enable high-energy X-ray techniques such as powder diffraction, pair distribution function (PDF) analysis, small-angle X-ray scattering (SAXS), X-ray fluorescence (XRF), and X-ray absorption spectroscopy (XAS)^44^ to be applied to complex catalyst systems. Correlative and multimodal approaches^45–47^ provide complementary insight into crystallinity, domain size, strain, phase composition (diffraction), particle morphology, density, and arrangement independent of crystallinity (SAXS), chemistry (XRF), and local chemical environment and oxidation state (XAS). PDF analysis further extends structural sensitivity to disordered and amorphous components, provided that sufficient signal-to-background ratios are achieved over a wide scattering vector range.^35,48^ In addition, X-ray photon correlation spectroscopy sheds light on motions within the electrolyte.^49,50^ While these techniques are individually well established and increasingly combined, fully quantitative analyses—such as extracting information from diffuse scattering between Bragg peaks^51^ or X-ray Coulomb counting^52^—remain underutilized. Importantly, diffuse scattering contains critical information on thermal motion, disorder, and amorphous phase fractions, which are particularly relevant for understanding degradation processes in electrocatalysis, but is often simply treated as background intensity, which compromises the measurement.

An effective strategy for *operando* studies is to reduce the system complexity by employing supported nanoparticles or nanostructured thin films as model electrodes.^53^ This approach enables the use of surface-sensitive techniques such as grazing-incidence wide-angle (GIWAXS)^54^ and small-angle (GISAXS)^55^ X-ray scattering as well as X-ray reflectometry (XRR),^56^ while allowing a horizontal working electrode geometry.^35^ Such a configuration facilitates beam spreading, thereby reducing radiation damage, and enables lower catalyst loadings closer to those used in electrocatalytic measurements.^36^ In addition, horizontal geometries improve gas bubble removal and provide compatibility with complementary techniques,^57^ including surface spectroscopy, *operando* LP-EM, and scanning electrochemical microscopy.^36^ GIWAXS and GISAXS yield information analogous to powder diffraction and SAXS, respectively, while offering tunable surface sensitivity via adjustment of the incidence angle near the critical angle for total external reflection.^35^ However, challenges remain, including reduced angular resolution, an extended footprint in the beam direction, dynamical and refraction effects, and the limited availability of robust quantitative analysis routines. In particular, quantitative modeling^58^ of GIWAXS data is essential and requires further methodological development to enable a comprehensive understanding of the catalyst degradation.

Finally, combining high-energy X-ray methods with complementary spectroscopic techniques, such as infrared absorption spectroscopy,^59^ offers additional insight into electrocatalyst behavior under *operando* conditions. While synchrotron-based measurements provide superior time resolution, laboratory-based high-energy X-ray setups^48,60,61^ offer greater accessibility for routine studies. Although typically limited in temporal resolution, such measurements are well-suited for probing long-term processes, including degradation pathways, and can effectively complement synchrotron experiments by guiding experimental design and maximizing the information gained from limited beam time. Overall, *operando* X-ray studies are well-suited to complement *operando* LP-EM experiments in electrocatalysis via providing statistical benchmarks of larger sample volumes up to small devices.^57^

## Scanning Electrochemical Probe Microscopy

While *operando* LP-EM and X-ray techniques provide valuable insights into structural evolution, compositional changes, electronic structure, and oxidation states, scanning electrochemical probe microscopies (SEPMs) offer a complementary toolbox for investigating spatially resolved electrochemical activity and degradation processes at model electrode–electrolyte interfaces. For degradation studies, failure mechanisms typically originate at defect sites, grain boundaries, facets, and film–substrate interfaces. SEPMs map local electrochemical reactivity to relate catalytic performance with surface or structural heterogeneities. By resolving the local heterogeneities, SEPMs identify the degradation “hotspots” that are hidden in ensemble-averaged electrochemical measurements.^62^

Electrochemical atomic force microscopy (EC-AFM) operates based on optical detection of mechanical deflection of a cantilever (typical diameters 10–100 nm) arisen from the tip–substrate interaction.^63^ Among the SEPMs, EC-AFM offers the highest spatial resolution for live tracking topographical changes, electrostatic properties, mechanical properties (adhesion, deformation, etc.) under *operando* electrochemical conditions.^64^ EC-AFM track degradation processes such as surface restructuring and particle dissolution, allowing direct correlation with applied potential under reaction conditions.

In contrast to EC-AFM, scanning electrochemical microscopy (SECM) probes local electrochemical activity through the detection of faradaic or nonfaradaic currents from the conversion of freely diffusing redox species at its ultra-microelectrode (UME, typical diameters 10–100 μm) tip.^62^ The measured tip current largely depends on the tip–substrate distance and local interfacial reactivity, as the substrate can influence the diffusion by hindering, consuming, or generating species. The potential at the tip and substrate can be independently biased, and through the different modes (e.g., feedback, generation-collection, redox competition, or direct), SECM probes local electron transfer kinetics, concentrations, and fluxes of reactants, intermediates, or products, under *operando* conditions. These factors are critical for understanding degradation phenomena, which may involve pH variations, formation or depletion of (intermediate) reactants, increased overpotentials, or inhomogeneous current distribution.

In EC-AFM and SECM systems, the electrolyte covers the substrate in a blanket manner, whereas scanning electrochemical cell microscopy (SECCM) employs a filled micro-/nanopipette (typical diameters 100 nm to 1 μm)^65^ forming a confined meniscus droplet at the substrate similar to scanning flow-cell geometries (cf. SFC-ICP-MS), with a small contact area that is slightly larger than the pipet opening. The “on-demand” droplet cell offers flexibility to perform measurements on broader types of substrates, including direct imaging on TEM grids.^66^ Importantly, the electrolyte is freshly delivered via the nanopipette to each spot. In other words, the spot has not been exposed to the electrolyte prior to the approach of nanopipette, thereby avoiding possible preinteraction between the substrate and the electrolyte (cross talk) as for the case of SECM and EC-AFM. The existing spatial resolution and sensitivity of SECCM enables probing electrochemical activity and degradation at the nanoscale, allowing differentiation between grains and grain boundaries, identification of defects, facet- or edge-dependent reactivity in micro- and nanostructures.

Despite their powerful capabilities, SEPMs possess inherent limitations, particularly arising from their operation in a liquid environment. In EC-AFM, mechanical interactions between the tip and surface may modify the surface, such as introducing artifacts or altering the degradation path. Prolonged measurements may lead to tip blunting, wear-off, or contamination, contributing to tip-induced artifacts such as broadening or distortion of surface features. Furthermore, instrumental drift may cause motion or misalignment, limiting long-term, high-resolution imaging.^67^

In SECM, the UME tip modifies the local diffusion field, particularly at small tip–substrate separation, when the feedback effects become significant. In SECCM, the confined meniscus defines a highly localized electrolyte volume, therefore, reactant supply, product accumulation, and transport characteristics differ from bulk conditions, and are influenced by the droplet size. As a result, the current response measured by SECM and SECCM may include catalytic activities, degradation, and probe-induced modifications of the local environment. For instance, local pH mapping and finite element simulations have jointly shown that UME can alter local concentration and pH profiles near the electrocatalyst interface.^68^ Additionally, in SECM, the measured current may include both the surface reactivity and tip–substrate distance, leading to convolution of electrochemical and topographical contributions.

Furthermore, local electrochemical processes involve mass transfer and diffusion in the electrolyte. Consumption or generation of species at a spot creates concentration gradients that spread out beyond the size of the probed spot, leading to lateral cross-talk with neighboring spots. This effect is pronounced at high current densities or for slowly diffusing species, where mass transport limitations broaden the concentration fields. The spatial resolution is therefore larger than the probed area, which is typically determined by the probe size.

To improve these situations, careful control of tip–substrate distance of SECM or combining with scanning ion conductance microscopy (SICM) to maintain constant distance and reduce the convolution of topography-reactivity signals. In SECCM, the footprints of droplets are typically checked, and the hopping mode where the droplet is fully detached and repositioned to increase spatial separation allows relaxation of concentration gradients reducing lateral crosstalk.

Besides spatial limitation, in certain cases where degradation processes occur on time scales faster than SEPM scanning, dynamic behavior may not be fully captured. As surface mapping, especially for high-resolution imaging, requires considerable acquisition times, highly dynamic processes may evolve during measurement, leading to temporal averaging.

SEPM measurements are inherently localized, typically probing small model surface areas with relatively low throughput, such as LP-EM. This may raise concerns about the statistical relevance of the observed activity or degradation behavior, particularly for heterogeneous catalysts. Nevertheless, the localized droplet cell configuration in SECCM has demonstrated its potential in high-throughput measurements across predefined arrays with hundreds of locations, enabling efficient screening of larger surfaces. Such approaches are expected to improve mapping density and increase data statistics for correlations between local activity and macroscopic electrode (substrate) activity.

SEPMs’ ability to simultaneously probe local activity, interfacial chemistry, and structural evolution under *operando* conditions, distinguishes them from conventional electrochemical measurements.^62^ Nevertheless, the understanding of electrocatalyst degradation is not complete without composition, chemical, or electronic information. This motivates the development of hybrid SEPM techniques such as SECM-Raman spectroscopy,^69^ SECM-IR (infrared),^70^ AFM-SECM,^71^ SECCM-IRM (interference reflection microscopy),^72^ and SECCM-MS.^73^ These hybrid systems enable direct correlation between local electrochemical response and chemical or structural transformations at identical locations, although they are not yet widely applied in electrocatalysis degradation studies.

Importantly, the spatial scales of SEPM techniques, that is, the scanned areas of EC-AFM and SECM (ranging from ≈100 × 100 nm^2^ to 100 × 100 μm^2^) and confined droplet size in SECCM (typically area with ≈100–500 nm diameter) are comparable to many advanced microscopy and spectroscopy methods. This compatibility facilitates correlative workflows where the same active site, grain boundary, defect region, or degradation hotspot can be interrogated to obtain a full picture of the multidimensional, underlying cause and mechanism of degradation processes in electrocatalysis reactions, contributing to design or engineering of durable electrode material and electrocatalytic devices.

## Correlative Microscopy Approaches Involving *Operando* Methods for Disentangling Dynamic Electrocatalytic Processes

Recent *operando* studies presented the importance of correlative microscopy and spectroscopy for disentangling dynamic electrocatalyst evolution under reaction conditions (Figure 1). For the oxygen evolution reaction (OER), single crystalline β-Co­(OH)_2_ platelets experienced potential-dependent transformation in the structure and oxidation state. The platelets expanded in height and to α-CoO_2_H_1_._5_·0.5H_2_O like due to hydroxide intercalation pre-OER-potentials and contracted to initial height and to β-CoOOH again due to deintercalation of the protons in OER catalyzed by Co^3+^, at the right applied potentials. The height changes and the transformation of Co^2+^ ↔ Co^3+^ were monitored under correlative *operando* scanning probe and X-ray microscopy techniques, respectively. Despite the bulk structural transformation, under OER potentials, SECCM observed that the electrochemical current is largely confined to the edges instead of on the basal plane of the platelet, and X-ray microscopy and spectroscopy further recorded high Co^3+^ density at those reactive edges. These observations demonstrate the differences between where the current flows and where the bulk transforms.^74^

**Figure 1:**
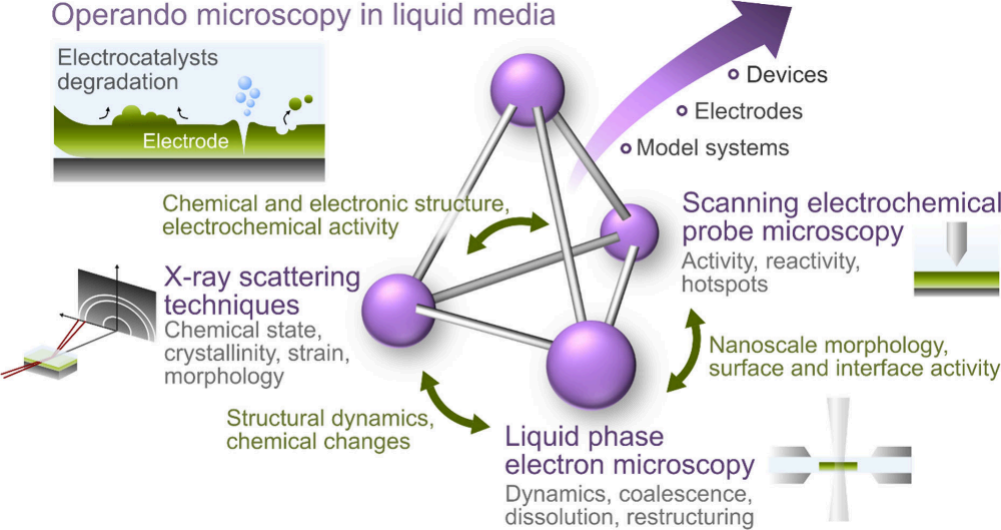
Embedding *operando* microscopy approaches into multiscale, multimodal, correlative electrochemical workflows.

Adding the temporal component, chronological and high-resolution tracking of the full life cycle and dynamic structural evolution of Cu nanocatalysts as small as ∼7 nm was achieved by *operando* methods under reaction conditions during the carbon dioxide reduction reaction (CO_2_RR). *Operando* liquid phase STEM directly visualized the dynamic restructuring of the initial Cu nanoparticle ensemble into nanoscale metallic Cu grains under reaction conditions, followed by transformation into single-crystalline Cu_2_O nanocubes upon postelectrolysis air exposure. Furthermore, *operando* 4D-STEM and high-energy-resolution and time-resolved X-ray spectroscopy demonstrated that the metallic Cu is rich in nanograin boundaries, providing undercoordinated active sites for selective reduction of CO_2_ to C_2+_ products.^75^

The kinetics study was extended to the nitrate reduction reaction (NO_3_RR). *Operando* EC liquid phase EM morphology and *operando* transmission soft X-ray microscopy, hard X-ray absorption spectroscopy, and Raman spectroscopy jointly showed that Cu_2_O nanocubes form potential-, time- and electrolyte-dependent restructuring and chemical state changes. Under moderate reductive conditions, Cu_2_O can be kinetically stabilized for longer periods via surface hydroxide formation that delays its reduction to metallic Cu. Further NH_3_ production alkalized the electrolyte and destabilized the hydroxide layer, inevitably forming soluble Cu complexes that drive catalyst restructuring through dissolution–redeposition and nanoparticle aggregation.^57^

Although these studies are not explicitly framed around degradation mechanisms, they establish a mechanistic foundation for *operando* degradation by exploring the heterogeneity of catalytic activities and dynamic restructuring, evidencing that correlative *operando* approaches (Figure 1) can be extended to track long-term irreversible degradations and reduced performance. Moreover, integrating multiple *operando* methods into characterization workflows can help overcome remaining drawbacks like, e.g., quantifying differences between irradiated and nonirradiated systems, which was recently shown for electrochemical LP-EM and SECCM.^76^

## Conclusions and Outlook

Overall, probe–sample interaction remains an inherent issue in all *operando* microscopies, which, however, can be addressed employing dedicated modeling and mitigation strategies. Moreover, embedding *operando* methods into correlative workflows harnessing *ex situ* techniques where needed can be used to largely exclude the impact of probe effects on data and their interpretation. Correlative *operando* LP-EM, X-ray, and electrochemical probe-based analyses provide a powerful framework for scale-bridging and multimodal investigations of electrocatalytic degradation processes. Each of these techniques offers distinct strengths, but also inherent limitations: LP-EM provides spatially resolved insight into local structural dynamics yet may suffer from beam-induced effects and limited statistical relevance; *operando* X-ray methods offer statistically robust structural and chemical information under more realistic reaction environments but are typically spatially averaged; and SEPM enables spatially resolved electrochemical activity mapping, while lacking direct access to the underlying structural evolution. By combining these complementary approaches, the strengths of each method can be exploited, while their individual weaknesses can be cross-validated or deconvolved. For example, small-scale LP-EM observations can be benchmarked against statistically representative *operando* X-ray data, while mass-transport-related phenomena may be identified or excluded by comparison to more realistic X-ray model reactors. Similarly, SEPM scans of electrodes subsequently analyzed by LP-EM can link local electrochemical activity to structural information, whereas *post-mortem* SEPM may help to quantify beam-induced artifacts. Furthermore, combining SEPM with surface-sensitive operando X-ray methods allows spatially averaged structural and chemical changes to be correlated with electrochemical activity and degradation at specific sites. In this way, the targeted use of correlative *operando* techniques can unfold the full potential of the individual tools, enabling access to transient states, kinetics, and dynamic processes that are essential for disentangling degradation mechanisms in electrocatalysis. Complementary multimodal *ex situ* characterization can further strengthen working hypotheses by providing high-resolution structural and chemical information, for example, through identical-location analyses before and after electrochemical testing. Such correlative approaches are necessary for scaling bridging atomistic processes to ensemble behavior in time and space. Furthermore, they enable bridging the gap between model systems and real devices. Making this happen, however, requires a common language for experts from different fields of methodology to communicate effectively, which appears to be one of the remaining main hurdles. A dedicated, multidisciplinary education comprising methods, materials, and data science could, therefore, enable realization of accelerated materials development by employing more effective characterization workflows for analyzing hierarchical devices with ever increasing complexity.
